# Supplementary material for: The Impact of Heavy Metal Accumulation on Some Physiological Parameters in Silphium perfoliatum L. Plants Grown in Hydroponic Systems
Source: Plants (Basel). 2023 Apr 20;12(8):1718. doi: 10.3390/plants12081718 (PMC10146597; doi:10.3390/plants12081718)
Supplement: Supplementary file 1 [file plants-12-01718-s001.zip › plants-2312368-supplementary.pdf]

Table S1. Test the homogeneity of variance for heavy metal concentrations in different organs of *S. perfoliatum* plants grown under different treatments.

Treatment	Test	Metal			
		Cu	Zn	Cd	Pb
Control	Cochran's C	0.431; p=0.971	0.556; p=0.592	0.769; p=0.161	0.832; p=0.085
	Bartlett's	1.05; p=0.886	1.115; p=0.766	2.596; p=0.096	1.848; p=0.221
Cu	Cochran's C	0.417; p=1.00	0.772; p=0.155	0.667; p=0.332	0.789; p=0.134
400 ppm	Bartlett's	1.035; p=0.919	2.685; p=0.088	1.511; p=0.363	2.195; p=0.145
Zn	Cochran's C	0.781; p=0.142	0.813; p=0.117	0.735; p=0.211	0.891; p=0.074
1200 ppm	Bartlett's	2.657; p=0.117	2.257; p= 0.175	1.448; p=0.403	3.242; p=0.065
Cd	Cochran's C	0.837; p=0.079	0.515; p=0.706	0.551; p=0.605	0.478; p=0.817
20 ppm	Bartlett's	1.993; p=0.184	1.089; p=0.811	1.349; p=0.749	1.549; p=0.341
Pb	Cochran's C	0.814; p=0.115	0.446; p=0.920	0.504; p=0.738	0.973; p=0.045
400 ppm	Bartlett's	3.015; p=0.082	1.076; p=0.836	1.217; p=0.617	4.541; p=0.018
Cu+Zn+	Cochran's C	0.546; p=0.619	0.514; p =0.709	0.603; p=0.703	0.872; p=0.083
Cd+Pb	Bartlett's	2.154; p=0.152	1.089; p=0.811	0.945; p=0.655	3.281; p=0.062

Table S2. ANOVA for heavy metal concentrations in different organs of *S. perfoliatum* plants grown under different treatments.

Treatment	Source		Cu		Zn		Cd		Pb	
	of variation	DF	MS	F	MS	F	MS	F	MS	F
Control	Replications	4	0.54	1.30	8.79	0.84	0.038	2.50	0.02	1.60
	Plant organs	2	14.50	35.27**	209.22	20.10**	2.753	183.51**	1.181	94.50**
	Residual	8	0.41		10.41		0.015		0.013	
Cu	Replications	4	986	2.07	11.25	2.23	0.29	1.35	2.25	1.62
400 ppm	Plant organs	2	212163	445.95**	3599.46	714.53**	48.30	227.30**	238.43	171.84**
	Residual	8	476		5.04		0.21		1.39	
Zn	Replications	4	1.50	1.40	73.50	1.16	0.31	1.30	63.75	1.05
1200 ppm	Plant organs	2	228.25	212.32**	1819.01	28.69**	23.54	98.59**	38599.53	635.38**
	Residual	8	1.08		63.38		0.24		60.75	
Cd	Replications	4	4.25	1.68	21.50	1.73	128.75	1.89	0.11	1.41
20 ppm	Plant organs	2	570.50	225.94**	481.73	38.73**	14104.67	206.85**	8.13	106.62**
	Residual	8	2.53		12.44		68.19		0.08	
Pb	Replications	4	45.25	2.04	20.25	1.82	0.013	1.54	1350	2.24
400 ppm	Plant organs	2	18250.73	822.57**	270.50	24.31**	0.823	101.33**	3899572	6481**
	Residual	8	22.19		11.13		0.008		602	
Cu+Zn+	Replications	4	487.50	1.89	62.75	2.11	92.25	1.57	1299	1.27
Cd+Pb	Plant organs	2	68279.94	264.59**	1438.22	48.45**	15956.84	271.61**	1493731	1465.7**
	Residual	8	258.06		29.69		58.75		1019	

** Significant at $p \leq 0.01$;

Table S3. Test the homogeneity of variance for photosynthetic pigments in *S. perfoliatum* plants grown under different treatments.

Pigments	Cochran's C test	Bartlett's test
Chl a	0.390; p=0.692	1.195; p=0.829
Chl b	0.553; p=0.201	1.519; p=0.480
Chl a+b	0.409; p=0.609	1.241; p=0.773
Car	0.499; p=0.313	1.597; p=0.419
Total	0.359; p=0.842	1.255; p=0.756
Chl a/b	0.469; p=0.397	2.246; p=0.150
Chl a+b /Car	0.403; p=0.634	2.846; p=0.068
Chl a+b /Total	0.427; p=0.539	1.983; p=0.222
Car /Total	0.427; p=0.539	1.983; p=0.222

Chl-Chlorophyll; Car-Carotenoids

Table S4. ANOVA for photosynthetic pigments in *S. perfoliatum* plants grown under different treatments.

Source		Chl a		Chl b		Chl a+b		Car		Total	
of variation	DF	MS	F	MS	F	MS	F	MS	F	MS	F
Replications	4	4.58	2.02	0.51	2.37	7.86	2.20	0.23	1.95	8.36	1.89
Plant organs	4	36.02	15.92**	7.43	34.86**	65.20	18.27**	3.76	31.84**	78.55	17.81**
Residual	16	2.26		0.21		3.57		0.12		4.41	

** Significant at $p \leq 0.01$; Chl-Chlorophyll; Car-Carotenoids

Table S5. Test the homogeneity of variance for antioxidant enzymatic activities of superoxide dismutase (SOD), catalase (CAT) and peroxidase (POD) in different organs of *S. perfoliatum* plants under different treatments.

Treatment	Test	Antioxidant enzymatic activities		
		SOD	CAT	POD
Control	Cochran's C	0,429; p=0.977	0.721; p=0.233	0.579; p=0.532
	Bartlett's	1.039; p=0.911	1.416; p=0.426	1.289; p=0.536
Cu	Cochran's C	0.434; p=0.959	0.511; p=0.718	0.645; p=0.377
400 ppm	Bartlett's	1.025; p=0.942	1.401; p=0.437	1.233; p=0.598
Zn	Cochran's C	0.423; p=0.998	0.633; p=0.404	0.476; p=0.824
1200 ppm	Bartlett's	1.029; p=0.932	1.338; p=0.489	1.087; p=0.814
Cd	Cochran's C	0.517; p=0.699	0.421; p=1.0	0.491; p=0.781
20 ppm	Bartlett's	1.077; p=0.834	1.02; p=0.952	1.249; p=0.579
Pb	Cochran's C	0.478; p=0.816	0.456; p=0.887	0.527; p=0.672
400 ppm	Bartlett's	1.103; p=0.786	1.089; p=0.811	1.096, p=0.798
Roots	Cochran's C	0.377; p=0.752	0.363; p=0.824	0.331; p=1.0
	Bartlett's	1.156; p=0.877	1.088; p=0.950	1.140; p=0.895
Stems	Cochran's C	0.401; p=0.642	0.420; p=0.565	0.431; p=0.524
	Bartlett's	1.435; p=0.556	1.524; p=0.476	1.23; p=0.786
Leaves	Cochran's C	0.477; p=0.466	0.304; p=1.0	0.354; p=0.870
	Bartlett's	1.354; p=0.641	1.292; p=0.711	1.316; p=0.683

Table S6. ANOVA for antioxidant enzymatic activities of superoxide dismutase (SOD), catalase (CAT) and peroxidase (POD) in different organs of *S. perfoliatum* plants under different treatments.

**Treatment**	**Source of variation**	**DF**	**SOD**		**CAT**		**POD**	
			**MS**	**F**	**MS**	**F**	**MS**	**F**
Control	Replications	4	34.75	0.26	0.013	1.67	0.21	1.98
	Plant organs	2	202.01	1.53 ns	1.32	175.97**	29.12	274.09**
	Residual	8	132.25		0.008		0.11	
Cu 400 ppm	Replications	4	59.25	1.39	0.11	2.14	0.66	1.62
	Plant organs	2	383.89	9.03**	10.57	201.34**	91.60	226.17**
	Residual	8	42.50		0.05		0.41	
Zn 1200 ppm	Replications	4	144.5	1.78	0.10	1.71	0.85	1.47
	Plant organs	2	1780.15	21.88**	12.98	216.38**	61.44	105.93**
	Residual	8	81.38		0.06		0.58	
Cd 20 ppm	Replications	4	53.36	1.27	0.043	1.48	0.79	1.24
	Plant organs	2	3017.73	72.07**	0.989	34.41**	103.31	162.05**
	Residual	8	41.88		0.029		0.64	
Pb 400 ppm	Replications	4	104.5	1.51	0.063	1.85	0.35	2.12
	Plant organs	2	2226.23	32.15**	1.023	30.30**	18.59	112.68**
	Residual	8	69.25		0.034		0.17	
**Plant organ**	**Source of variation**	**DF**	**SOD MS**	**SOD F**	**CAT MS**	**CAT F**	**POD MS**	**POD F**
Roots	Replications	4	94.75	1.28	0.033	1.86	0.57	1.36
	Plant organs	4	6815.63	92.42**	10.55	602.99**	50.86	121.46**
	Residual	16	73.75		0.018		0.42	
Stems	Replications	4	162.25	1.73	0.078	1.43	0.60	1.10
	Plant organs	4	5588.04	59.69**	26.64	489.96**	165.83	304.98**
	Residual	16	93.63		0.054		0.54	
Leaves	Replications	4	112.25	2.15	0.047	1.30	0.20	1.17
	Plant organs	4	1850.55	35.37**	4.73	130.61**	31.57	187.77**
	Residual	16	52.31		0.036		0.17	

** Significant at $p \leq 0.01$; ns- non-significant.

Table S7. Variance components of multiple regressions between antioxidant enzymatic activity of superoxide dismutase (SOD) and heavy metal concentrations in organs of *S. perfoliatum* plants

Source of variation	SS	DF	MS	F
Roots				
Regression	9118.2	4	2279.55	46.62**
Cu (x_1)	230.79	1	230.79	4.72
Zn (x_2)	290.33	1	290.33	5.94*
Cd (x_3)	6182.08	1	6182.08	126.43**
Pb (x_4)	2415.01	1	2415.01	49.39**
Residual	488.96	10	48.90	
Total	9607.18	14		
$\boldsymbol{y} = 207.57 - 0.07x_1 - 0.24x_2 - 0.38x_3 - 0.02x_4$				
R^2 =0.9491; R^2_a =0.9287; SEE = 6.99; DW=2.19				
Source of variation	SS	DF	MS	F
Stems				
Regression	7680.4	4	1920.1	39.51**
Cu (x_1)	587.84	1	587.84	12.10**
Zn (x_2)	1824.89	1	1824.89	37.55**
Cd (x_3)	5208.73	1	5208.73	107.18**
Pb (x_4)	58.93	1	58.93	1.21
Residual	485.99	10	48.60	
Total	8166.38	14		
$y = 229.637 + 0.002x_1 - 0.648x_2 - 0.363x_3 - 0.184x_4$				
R^2 =0.9405; R^2_a =0.9167; SEE = 6.97; DW=2.47				
Source of variation	SS	DF	MS	F
Leaves				
Regression	2564.4	4	641.1	19.33**
Cu (x_1)	634.24	1	634.24	19.12**
Zn (x_2)	203.18	1	203.18	6.13**
Cd (x_3)	4.37	1	4.37	0.13
Pb (x_4)	1722.63	1	1722.63	51.94**
Residual	331.64	10	33.16	
Total	2896.06	14		
$y = 206.09 - 0.09x_1 + 0.13x_2 - 0.58x_3 - 0.58x_4$				
R^2 =0.8855; R^2_a =0.8397; SEE = 5.76; DW=2.69				

* Significant at $p \le 0.05$; ** Significant at $p \le 0.01$.
SEE - Standard error of estimate; DW - Durbin Watson statistic.

Table S8. Variance components of multiple regressions between antioxidant enzymatic activity of catalase (CAT) and heavy metal concentrations in organs of *S. perfoliatum* plants

Source of variation	SS	DF	MS	F
Root s				
Regression	14.032	4	3.508	201.42**
Cu (x_1)	0.040	1	0.040	2.32
Zn (x_2)	1.145	1	1.145	65.76**
Cd (x_3)	10.754	1	10.754	617.45**
Pb (x_4)	2.093	1	2.093	120.15**
Residual	0.174	10	0.017	
Total	14.207	14		
$y = 0.0181 + 0.0013x_1 + 0.0199x_2 + 0.0159x_3 + 0.0006x_4$				
$R^2 = 0.9877$; $R^2_a = 0.9828$; SEE = 0.132; DW=2.20				
Source of variation	SS	DF	MS	F
Stems				
Regression	34.571	4	8.643	64.31**
Cu (x_1)	4.957	1	4.957	36.88**
Zn (x_2)	22.329	1	22.329	166.15**
Cd (x_3)	7.251	1	7.251	53.95**
Pb (x_4)	0.035	1	0.035	0.26
Residual	1.344	10	0.134	
Total	35.915	14		
$y = -1.0254 - 0.0005x_1 + 0.0503x_2 + 0.0133x_3 + 0.0045x_4$				
$R^2 = 0.9626$; $R^2_a = 0.9476$; SEE = 0.367; DW=2.40				
Source of variation	SS	DF	MS	F t
Leaves				
Regression	6.281	4	1.570	54.51**
Cu (x_1)	0.103	1	0.103	3.57
Zn (x_2)	5.035	1	5.035	174.79**
Cd (x_3)	0.373	1	0.373	12.96**
Pb (x_4)	0.769	1	0.769	26.70**
Residual	0.288	10	0.029	
Total	6.569	14		
$y = 0.811 - 0.003x_1 + 0.044x_2 - 0.025x_3 - 0.012x_4$				
$R^2 = 0.9562$; $R^2_a = 0.9386$; SEE = 0.169; DW=2.15				

* Significant at $p \leq 0.05$; ** Significant at $p \leq 0.01$;
SEE - Standard error of estimate; DW - Durbin Watson statistic.

Table S9. Variance components of multiple regressions between antioxidant enzymatic activity of peroxidase (POD) and heavy metal concentrations in organs of *S. perfoliatum* plants

Source of variation	SS	DF	MS	F
Roots				
Regression	68.970	4	17.243	93.85**
Cu (x_1)	8.985	1	8.985	48.91**
Zn (x_2)	0.703	1	0.703	3.83
Cd (x_3)	46.654	1	46.654	253.94**
Pb (x_4)	12.629	1	12.629	68.74**
Residual	1.837	10	0.184	
Total	70.808	14		
$y = 5.8459 - 0.0004x_1 + 0.0819x_2 + 0.0319x_3 - 0.0014x_4$				
R^2 =0.9740; R^2_a =0.9637; SEE = 0.428; DW=2.69				
Source of variation	SS	DF	MS	F
Stems				
Regression	222.010	4	55.503	198.24**
Cu (x_1)	78.198	1	78.198	279.31**
Zn (x_2)	38.654	1	38.654	138.07**
Cd (x_3)	96.534	1	96.534	344.80**
Pb (x_4)	8.624	1	8.624	30.80**
Residual	2.800	10	0.280	
Total	224.810	14		
$y = -3.207 + 0.003x_1 + 0.086x_2 + 0.04x_3 - 0.07x_4$				
R^2 =0.9875; R^2_a =0.9826; SEE = 0.529; DW=3.17				
Source of variation	SS	DF	MS	F
Leaves				
Regression	41.527	4	10.382	60.23**
Cu (x_1)	12.255	1	12.255	71.09**
Zn (x_2)	28.271	1	28.271	164.00**
Cd (x_3)	0.008	1	0.008	0.05
Pb (x_4)	0.993	1	0.993	5.76*
Residual	1.724	10	0.172	
Total	43.251	14		
$y = -0.036 + 0.003x_1 + 0.073x_2 + 0.013x_3 + 0.014x_4$				
R^2 =0.9601; R^2_a =0.9442; SEE = 0.415; DW=2.31				

* Significant at $p \leq 0.05$; ** Significant at $p \leq 0.01$;
SEE - Standard error of estimate; DW - Durbin Watson statistic.
